# Effects of gestational age on brain volume and cognitive functions in generally healthy very preterm born children during school-age: A voxel-based morphometry study

**DOI:** 10.1371/journal.pone.0183519

**Published:** 2017-08-29

**Authors:** Sakari Lemola, Nadine Oser, Natalie Urfer-Maurer, Serge Brand, Edith Holsboer-Trachsler, Nina Bechtel, Alexander Grob, Peter Weber, Alexandre N. Datta

**Affiliations:** 1 Department of Psychology, University of Warwick, Coventry, United Kingdom; 2 Division of Neuropediatrics and Developmental Medicine, University of Basel, Children’s Hospital Basel, Basel, Switzerland; 3 Department of Psychology, University of Basel, Basel, Switzerland; 4 Center for Affective, Stress and Sleep Disorders (ZASS), Psychiatric Clinics (UPK), University of Basel, Basel, Switzerland; 5 Department of Sport, Exercise and Health, Division of Sport and Psychosocial Health, Faculty of Medicine, University of Basel, Basel, Switzerland; 6 Sleep Disorders Research Center, Kermanshah University of Medical Sciences (KUMS), Kermanshah, Iran; University Children's Hospital Tuebingen, GERMANY

## Abstract

**Objective:**

To determine whether the relationship of gestational age (GA) with brain volumes and cognitive functions is linear or whether it follows a threshold model in preterm and term born children during school-age.

**Study design:**

We studied 106 children (*M* = 10 years 1 month, *SD* = 16 months; 40 females) enrolled in primary school: 57 were healthy very preterm children (10 children born 24–27 completed weeks’ gestation (extremely preterm), 14 children born 28–29 completed weeks’ gestation, 19 children born 30–31 completed weeks’ gestation (very preterm), and 14 born 32 completed weeks’ gestation (moderately preterm)) all born appropriate for GA (AGA) and 49 term-born children. Neuroimaging involved voxel-based morphometry with the statistical parametric mapping software. Cognitive functions were assessed with the WISC-IV. General Linear Models and multiple regressions were conducted controlling age, sex, and maternal education.

**Results:**

Compared to groups of children born 30 completed weeks’ gestation and later, children born <28 completed weeks’ gestation had less gray matter volume (GMV) and white matter volume (WMV) and poorer cognitive functions including decreased full scale IQ, and processing speed. Differences in GMV partially mediated the relationship between GA and full scale IQ in preterm born children.

**Conclusions:**

In preterm children who are born AGA and without major complications GA is associated with brain volume and cognitive functions. In particular, decreased brain volume becomes evident in the extremely preterm group (born <28 completed weeks’ gestation). In preterm children born 30 completed weeks’ gestation and later the relationship of GA with brain volume and cognitive functions may be less strong as previously thought.

## Introduction

Very preterm birth is a risk for normal development of the brain [1,2] even in the absence of major perinatal complications such as periventricular leukomalacia (PVL), intraventricular haemorrhage (IVH), and periventricular haemorrhagic infarction (PHI) [3–6]. Compared to term-born children, very preterm children had smaller brain volume including smaller gray matter volume (GMV) and white matter volume (WMV) in childhood [6–8], adolescence [9–12], and young adulthood [4,13]. Compared to their peers born at term, regional volume differences in very preterm children, adolescents, and young adults were wide spread and included decreases of GMV in all lobes (frontal [9,12,13,14], temporal [4,7,9,12,14], parietal [7,9] and occipital lobes [12,13]). Further, decreased GMV was observed in the hippocampus [9,11,15,16], thalamus [4,9,12,13,15], and cerebellum [12,17].

There is a large body of evidence showing that both gestational age [18] and brain volume are related to cognitive function [9,12]. With regard to regional GMV one study showed that in preterm children born between 30 and 34 weeks’ gestation and with low risk for neurologic deficit or developmental difficulties, GMV in the temporal lobe was significantly reduced, which in turn was related with decreased cognitive functions [19].

However, there is limited knowledge whether brain volume and cognitive function decrease linearly with decreasing gestational age (GA) or whether there is a threshold of GA above which the brain remains unaffected. Existing evidence on the association between gray matter volume (GMV), white matter volume (WMV) and GA is inconsistent. Some studies found a linear association of decreasing GMV and WMV with earlier GA [7,20], even in moderately preterm children [21–23]. By contrast, one large study [9] showed no relationship between birth weight and GMV and WMV above a birth weight of 1500g suggesting a threshold above which maturity at birth (i.e., GA or birth weight) is no longer related to later brain size. Possibly, studies indicating a linear relationship vs. studies indicating a threshold model of the relationship between GA and brain volume differed in sample composition. Such differences between studies may involve the prevalence of other neonatal risk factors in their samples such as the number of children with perinatal complications or children born small for gestational age (SGA), which may play an important role for later brain development [24]. With regard to the nature of the relationship between GA and cognitive function a recent study suggests the existence of a nonlinear relationship. In children from the Bavarian Longitudinal Study the association of GA with IQ and mathematic attainment became evident below a threshold of 34 weeks’ gestation while there was barely a relationship above this threshold [18].

The aim of the present study was to examine whether there is a linear decline in brain volume and cognitive functions with GA or whether the relationship is better described with a threshold model involving a stronger relationship of GMV, WMV, and cognitive functions with GA below a certain level of GA. Therefore, we compare five groups with different GA with each other, children born in the 24–27 completed weeks’ gestation (extremely preterm children), 28–29 completed weeks’ gestation (very preterm children, earlier group), 30–31 completed weeks’ gestation (very preterm children, later group), 32 completed weeks’ gestation (moderately preterm children), and term born children. This approach allows to describe brain and cognitive development in these subgroups and is therefore of interest for paediatricians, educational services, and parents of preterm children. Although these groups represent a considerable percentage of the newborn population in modern societies (in Germany [25] and the USA [26] respectively, children born 24–27 completed weeks’ gestation account for 0.24% and 0.49% of the newborn population, children born 28–29 completed weeks’ gestation account for 0.23% and 0.44%, children born 30–31 completed weeks’ gestation account for 0.36% and 0.76%, and children born 32 completed weeks’ gestation account for 0.30% and 0.59%), research comparing these subgroups of preterm children at school age regarding brain development is missing. To exclude effects of other perinatal risk factors, only children with low risk (i.e., without PVL, IVH, and PHI) and born appropriate for gestational age (AGA) were studied. Moreover, we studied whether differences in GMV accounted for differences in cognitive functions.

## Materials and methods

### Participants

Fifty-seven preterm children (24–32 completed weeks’ gestation; age: *M* = 10.0 years, *SD* = 1.3; range: 7.8 to 12.3) and 49 term born children (≥37 completed weeks’ gestation; age: *M* = 10.2 years, *SD* = 1.4; range: 7.9 to 12.8; t(104) = 0.87, *P* = 0.39) who successfully completed MRI scans were included in the present study. Descriptive statistics are presented in Table 1. Preterm children were recruited from an initial cohort of 515 children born 24–32 completed weeks’ gestation between January 1998 and December 2006 at the University Children’s Hospital Basel (Switzerland). Inclusion criteria were enrollment in regular primary school in Switzerland, no severe developmental delay, no evidence of major complications during the first year of life (i.e. exclusion of children with PVL, IVH of grade 2 or higher, and PHI), being born AGA (i.e., > 10th percentile of birth weight) [24], sufficient German language skills of the parents to give informed consent, and residence in Switzerland and within 100 km from the study center. Furthermore, because of MRI scanning, children with fixed dental braces were excluded. Of 62 preterm children who originally had MRI scanning, 5 were excluded due to movements during the MRI.

**Table 1:** Descriptive statistics for study variables (*N* = 106).

	Preterm children	Term born children	
	(*n* = 57)	(*n* = 49)	
	*M / N (SD / %)*	*M / N (SD / %)*	*P*
**Child age (years)**	10.02 (±1.25)	10.25 (±1.41)	0.386
**Sex (male)**	34 (65.3%)	32 (59.6%)	0.55
**Maternal education**^2^			
• **No vocational training**	12 (21.1%)	0 (0.0%)	<0.001
• **Vocational training**	33 (57.9%)	19 (38.8%)	
• **University**	10 (17.5%)	21 (42.9%)	
• **Information missing**	2 (3.5%)	9 (18.4%)	
**Birth weight (g)**	1447 (±427)	3320 (±541)	<0.001
**GMV (ml)**	777 (±70)	798 (±67)	0.112
**WMV (ml)**	464 (±67)	476 (±57)	0.301
**CSF (ml)**	157 (±30)	153 (±24)	0.533
**Total ICV (ml)**	1397 (±148)	1428 (±126)	0.256
**Full scale IQ (WISC-IV)**^1^	102.6 (±13.1)	107.8 (±10.2)	0.031
**Verbal Comprehension**^1^	102.7 (±14.0)	104.4 (±13.5)	0.554
**Reasoning**^1^	106.6 (±13.0)	112.2 (±8.6)	0.017
**Working Memory**^1^	103.8 (±12.9)	105.4 (±13.2)	0.552
**Processing speed**^1^	96.3 (±13.4)	101.9 (±11.6)	0.033

^1^ IQ score normative mean = 100 (SD = 15). GMV, Gray Matter Volume; WMV, White Matter Volume; CSF, Cerebrospinal Fluid; Total ICV, Total Intracranial Volume.

^2^ Maternal education is available for 95 children.

Compared to non-participants, the 57 preterm children with successful MRI scans had modestly higher birth weight (1447g vs. 1286g, *F*(1, 512) = 6.07; *P* = 0.01) and higher GA (29.7 weeks vs. 29.1 completed weeks, *F*(1, 514) = 4.36; *P* = 0.04); gender did not differ (*χ*^*2*^(1) = 0.01; *P* = 0.93). The participating preterm children included 10 children born 24–27 completed weeks’ gestation, 14 children born 28–29 completed weeks’ gestation, 19 children born 30–31 completed weeks’ gestation, and 14 born 32 completed weeks’ gestation (see Table 1 for participants’ characteristics). No child had PVL or PHI, while one child born 31 completed weeks’ gestation was diagnosed with mild IVH (grade 1).

Among the term born children three were excluded because of structural anomalies and six due to movement during the MRI scanning. The term born control group finally consisted of 49 children who were recruited from official birth notifications (n = 35; 71.4% of the control group), children from hospital staff (n = 4; 8.2% of the control group), children attending Children’s University (n = 2; 4.1% of the control group), one sibling of a preterm child (n = 1; 2.0% of the control group), healthy siblings of participants from another study (n = 3; 6.1% of the control group), and headache patients without structural abnormalities (n = 4; 8.2% of the control group). Parents gave written informed consent for the children to participate and assent was obtained from the child. The study was approved by the Ethics Committee of Basel.

### Procedure

Children visited the University Children's Hospital Basel (Switzerland) for neuroimaging and cognitive assessment was conducted by trained study personnel at the study center or at the children’s homes. Mothers completed questionnaires to assess demographic data (no vocational training, vocational training, university).

### Measurement

#### Cognitive assessment

Cognitive functions were assessed using the German version of the WISC-IV [27] which provides a full scale IQ representing a child’s global intellectual functioning, as well as four index scores representing specific cognitive abilities: verbal comprehension, perceptual reasoning, working memory, and processing speed. The full scale IQ and the index scores have a mean of 100 and a standard deviation of 15.

#### Neuroimaging procedure

Imaging of structural data was acquired using a 3-Tesla MRI with a standard head coil (Magnetom VERIO, Siemens Healthcare, Erlangen, Germany). Structural imaging was conducted with sagittal T1-weighted 3D high-resolution magnetization prepared rapid gradient echo sequence (MPRAGE), with TR = 2000 ms, TE = 3,4 ms, TI = 1000 ms and an isotropic spatial resolution of 1x1x1 mm^3^.

Image processing of voxel-based morphometric (VBM) analysis was conducted with the statistical parametric mapping software (SPM8) (Wellcome Department of Imaging Neuroscience, UCL Institute of Neurology, London, UK; http://www.fil.ion.ucl.ac.uk/spm/software/spm8/) implemented in Matlab (Version 7.9.0, Mathworks Inc., USA). Structural images were segmented into GMV and WMV, cerebrospinal fluid, bone, soft tissue and air/background with the new-segment tool in SPM8. Default settings were used. A customized age- and sex-matched Tissue Probability Map and a children’s T1-template using the average template approach of the Template-O-matic toolbox [28] was calculated to create an average template using the Diffeomorphic Anatomical Registration Through Exponentiated Lie Algebra method (DARTEL). Subsequently GMV and WMV images were warped to this average template and normalised into MNI space. Images were modulated non-linearly to correct for individual brain size. GMV images were then smoothed with 8mm Gaussian kernel, WMV images with 12mm. The quality check of the VBM8 toolbox (C. Gaser, University of Jena, Germany, VBM8-Toolbox Manual, 2010; http://dbm.neuro.uni-jena.de/vbm8/VBM8-Manual.pdf) was performed to check the accuracy of segmentation and normalization and to identify artefacts and outliers. The homogeneity check indicated no outliners above or below 2 standard deviations from the mean.

To examine regional GMV differences between groups, a full factorial design specification was assessed in SPM8 with the five groups. An absolute threshold mask of 0.3 was set to gray matter. All results were Family Wise Error (FWE) corrected at *p* = 0.05. Significant cluster of GMV were further analysed using the VOI toolbox in SPM8 to calculate regional volumes per child, again results were reported FWE corrected with a threshold of 0.05 in clusters > 30 voxel.

### Statistical analyses

Mean differences of brain volumes and cognitive functions between groups with varying GA were tested applying General Linear Models (GLM). First, multivariate GLM was conducted with GMV and WMV as dependent variables, “gestational age-groups” as fixed factor, and age, sex, and maternal education (dummy coded) as covariates (as potential further confounders we also analyzed the association of paternal education, household income, and number of children in the household with brain volume (GMV, WMV, CSF) and cognitive function (IQ, verbal comprehension, perceptual reasoning, working memory, processing speed) controlling age, sex, and maternal education; as none of these associations was significant (all p-values>0.05) only age, sex, and maternal education were used as covariates). Then polynomial tests and pairwise comparisons of the mean values of GMV and WMV between the five groups 24–27 completed weeks’ gestation, 28–29 completed weeks’ gestation, 30–31 completed weeks’ gestation, 32 completed weeks’ gestation, and term born children were conducted by bootstrapping based on 1000 bootstrap samples. Second, multivariate GLM was conducted with full scale IQ and the four index scores verbal comprehension, perceptual reasoning, working memory, and processing speed as dependent variables, gestational age-groups as fixed factor, and age, sex, and maternal education as covariates, which was again followed by polynomial tests and pairwise comparisons between the five groups. Finally, mediation analysis to examine whether GMV mediated the relationship between GA and cognitive functions was conducted according to Baron and Kenny [29] controlling age, sex, and maternal education among the preterm born children (24–32 completed weeks’ gestation). The nominal level of significance was set at alpha < .05. Statistical analyses were performed with SPSS^®^ 22.0 (IBM Corporation, Armonk NY, USA) for Apple Mac^®^.

## Results

### GMV, WMV, and cognitive differences in children with different GA at birth

**Fig 1:**
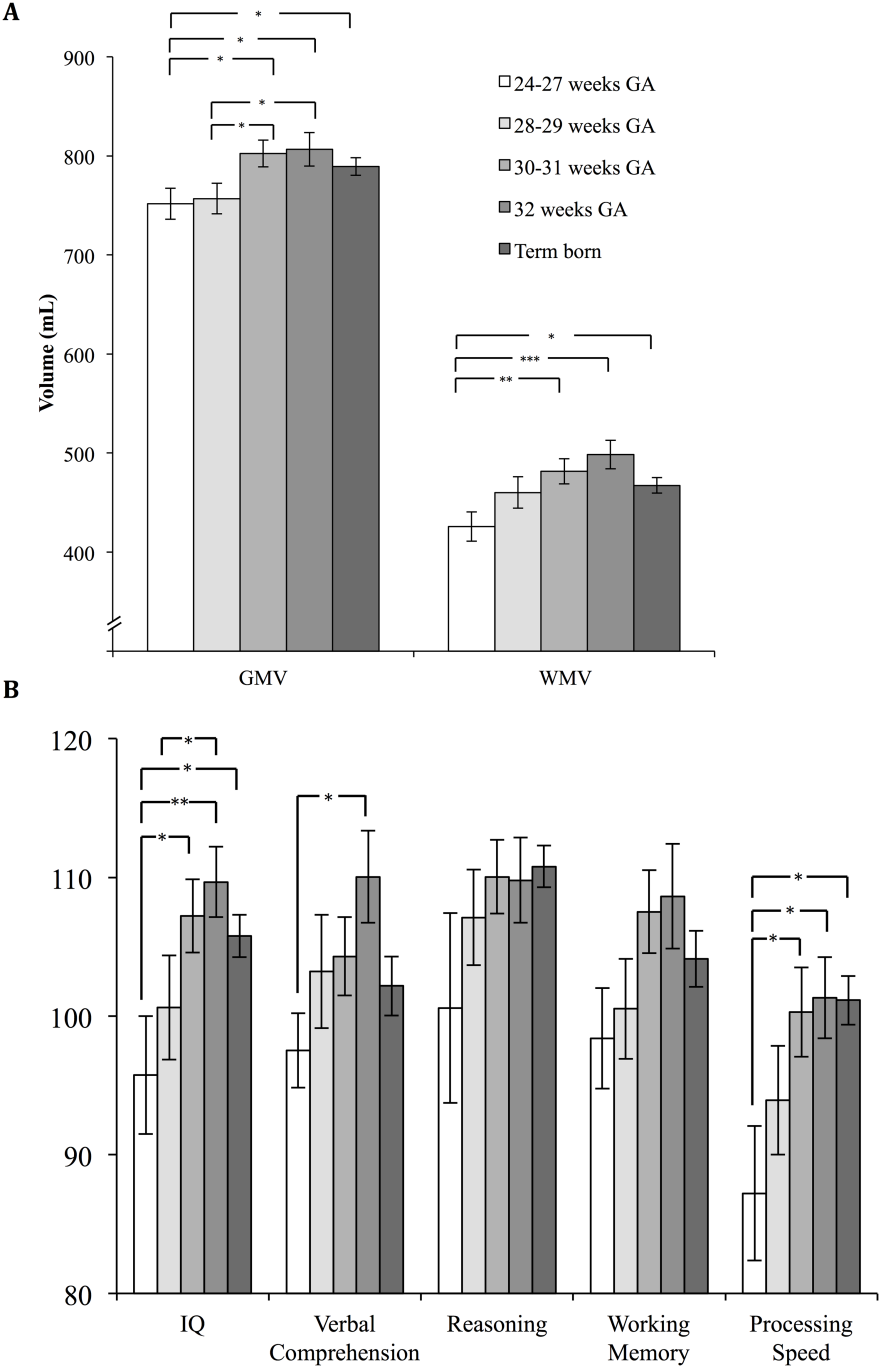
Brain volumes and cognitive functions by gestational age groups. **A)** Mean values and standard errors of Gray Matter Volume (GMV) and White Matter Volume (WMV) based on 1000 bootstrap samples by five gestational age groups (children born 24–27 completed weeks’ gestation, 28–29 completed weeks’ gestation, 30–31 completed weeks’ gestation, 32 completed weeks’ gestation and term born children) controlling age, sex, and maternal education. **B)** Mean values and standard errors of cognitive functions (IQ score normative mean = 100, SD = 15) based on 1000 bootstrap samples by five gestational age groups (children born 24–27 completed weeks’ gestation, 28–29 completed weeks’ gestation, 30–31 completed weeks’ gestation, 32 completed weeks’ gestation and term born children) controlling age, sex, and maternal education. * P < 0.05, ** P < 0.01, *** P < 0.001.

The multivariate GLM with GMV and WMV as dependent variables showed significant effects of the factors ‘groups of GA’ (*F*(4, 96) = 3.28, *p* = 0.015), age (*F*(2, 95) = 16.63, *p* < 0.001), sex (*F*(2, 95) = 17.01, *p* < 0.001), but not maternal education (*p*-values for all dummy variables > 0.05). Fig 1A and Table 2 present the brain volume differences in children with different GA at birth. Polynomial contrasts revealed a significant linear trend for GMV and WMV, as well as a significant quadratic trend for WMV (S1 Table, polynomial contrasts and pairwise comparisons between five gestational age groups (*P*-values) based on 1000 bootstrap samples and adjusted for age, sex, and maternal education). Pairwise comparisons showed that children born 24–27 completed weeks’ gestation and 28–29 completed weeks’ gestation had significantly smaller GMV compared to those born 30–31 completed weeks’ gestation and those born 32 completed weeks’ gestation. Children born 24–27 completed weeks’ gestation further differed significantly from term born children regarding GMV. Children born 24–27 completed weeks’ gestation had also significantly smaller WMV compared to the groups born 30–31 completed weeks’ gestation, 32 completed weeks’ gestation and term born children. The group born 30–31 completed weeks’ gestation and the group born 32 completed weeks’ gestation both showed non-significantly larger GMV and WMV than the term born group (all P-values >.10; Fig 1A, Table 2, S1 Table). Moreover, no significant differences in cerebrospinal fluid (CSF) volumes were found between the groups.

**Table 2:** Means and bias corrected bootstrap confidence intervals for five gestational age groups adjusted for age, sex, and maternal education.

	24–27 weeks	28–29 weeks	30–31 weeks	32 weeks	Term born
	*(n* = 10)	*(n* = 14)	*(n* = 19)	*(n* = 14)	*(n* = 49)
	*M (95%-CI)*	*M (95%-CI)*	*M (95%-CI)*	*M (95%-CI)*	*M (95%-CI)*
**GMV (ml)**	753 (720–782)	757 (725–788)	803 (777–829)	808 (770–843)	789 (773–807)
**WMV (ml)**	426 (396–455)	461 (430–493)	481 (457–506)	498 (471–529)	468 (453–482)
**CSF (ml)**	163 (141–185)	161 (149–175)	153 (139–171)	158 (149–168)	151 (145–159)
**Total ICV**	1343 (1264–1421)	1380 (1309–1454)	1438 (1385–1494)	1465 (1406–1527)	1407 (1375–1438)
**Full Scale IQ** ^1^	95.6 (85.8–104.7)	100.7 (93.1–108.4)	107.4 (102.2–113.6)	109.8 (104.3–114.9)	105.8 (102.8–108.6)
**Verbal Comprehension**^1^	97.5 (92.2–103.4)	103.5 (95.7–111.6)	104.5 (98.5–110.3)	109.9 (103.1–116.9)	102.1 (98.1–106.1)
**Reasoning**^1^	100.4 (85.3–114.0)	107.0 (100.3–114.1)	110.0 (104.6–115.2)	109.6 (103.8–115.2)	110.9 (107.6–113.8)
**Working Memory**^1^	98.5 (91.5–105.4)	100.4 (92.7–107.5)	107.5 (102.0–113.9)	109.0 (101.0–117.2)	104.3 (99.8–108.5)
**Processing speed**^1^	87.5 (77.8–97.9)	94.0 (85.1–102.8)	100.4 (93.8–107.5)	101.3 (95.3–107.7)	101.1 (97.8–104.4)

^1^ WISC-IV IQ score normative mean = 100 (SD = 15). GMV, Gray Matter Volume; WMV, White Matter Volume; CSF, Cerebrospinal fluid; ICV, Intracranial Volume.

The multivariate GLM with full scale IQ, verbal comprehension, perceptual reasoning, working memory, and processing speed as dependent variables showed significant effects of the factors ‘groups of GA’ (*F*(5, 86) = 3.07, *p* = 0.013), age (*F*(5, 83) = 3.01, *p* = 0.015), sex (*F*(5, 83) = 3.27, *p* = 0.010), and maternal tertiary education (*F*(5, 83) = 2.39, *p* = 0.045). Fig 1B and Table 2 present the differences regarding cognitive functions in children with different GA at birth. Polynomial contrasts revealed a significant linear trend and quadratic trend for full scale IQ and a significant linear trend for perceptual reasoning and processing speed (S1 Table). Pairwise comparisons showed that children born 24–27 completed weeks’ gestation had significantly lower full-scale IQ than the groups born 30–31 completed weeks’ gestation, 32 completed weeks’ gestation, and term born children. Moreover, children born 28–29 completed weeks’ gestation had significantly lower full-scale IQ than children born 32 completed weeks’ gestation. Regarding the four index scores representing specific cognitive abilities children born 24–27 completed weeks’ gestation had significantly lower processing speed than the groups born 30–31 completed weeks’ gestation, 32 completed weeks’ gestation, and term born children as well as lower verbal comprehension compared to the group born 32 completed weeks’ gestation. No significant differences were found between children born in the 30–31 completed weeks’ gestation and in the 32 completed weeks’ gestation compared to term born children regarding cognitive functions (all P-values >.10; Fig 1B, Table 2, S1 Table).

### Regional GMV differences in children with different GA at birth

**Fig 2:**
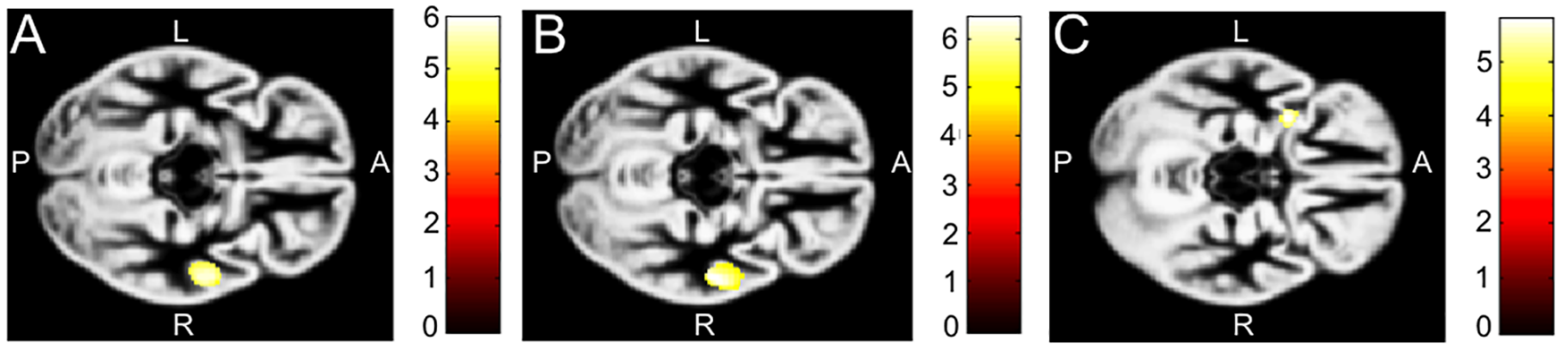
Regional differences of gray matter volume. Significant difference of GMV A) Contrast between children born 24–27 completed weeks’ gestation and term born children showing significantly decreased cortical GMV in one cluster in the right middle and superior temporal gyrus (MNI-coordinates: 56–6–15) with a cluster size of 542 voxel in the preterm group. B) Contrast between children born 28–29 completed weeks’ gestation and term born children showing significantly decreased cortical GMV in one cluster in the right middle and superior temporal gyrus (MNI-coordinates: 57–16–12), with a cluster size of 1487 voxel in the preterm group. C) Contrast between children born 32 completed weeks’ gestation and term born children showing significantly decreased cortical GMV in the left insula (MNI-coordinates: -32 6–15), with a cluster size of 145 voxel in the preterm group. The color bars represent the t-scores.

Fig 2 shows the analyses of regional cortical GMV differences. Compared to their term born peers, children born 24–27 completed weeks’ gestation as well as children born 28–29 completed weeks’ gestation had both decreased GMV in one cluster in the right middle and superior temporal gyrus (MNI-coordinates: 56, -6, -15; cluster size: 542 voxel; MNI-coordinates: 57, -16, -12; cluster size: 1487 voxel, respectively). While the children born 30–31 completed weeks’ gestation showed no significant differences from term born children, those born 32 completed weeks’ gestation had a decreased relative size of the left anterior insula (MNI-coordinates: -32, 6, -15; cluster size: 145 voxel). No regional differences were found between the groups 24–27 completed weeks’ gestation, 28–29 completed weeks’ gestation, 30–31 completed weeks’ gestation, and 32 completed weeks’ gestation controlling age, sex, and maternal education (Fig 2).

### Mediation of the relationship between preterm status and cognitive functions by brain volume

**Fig 3:**
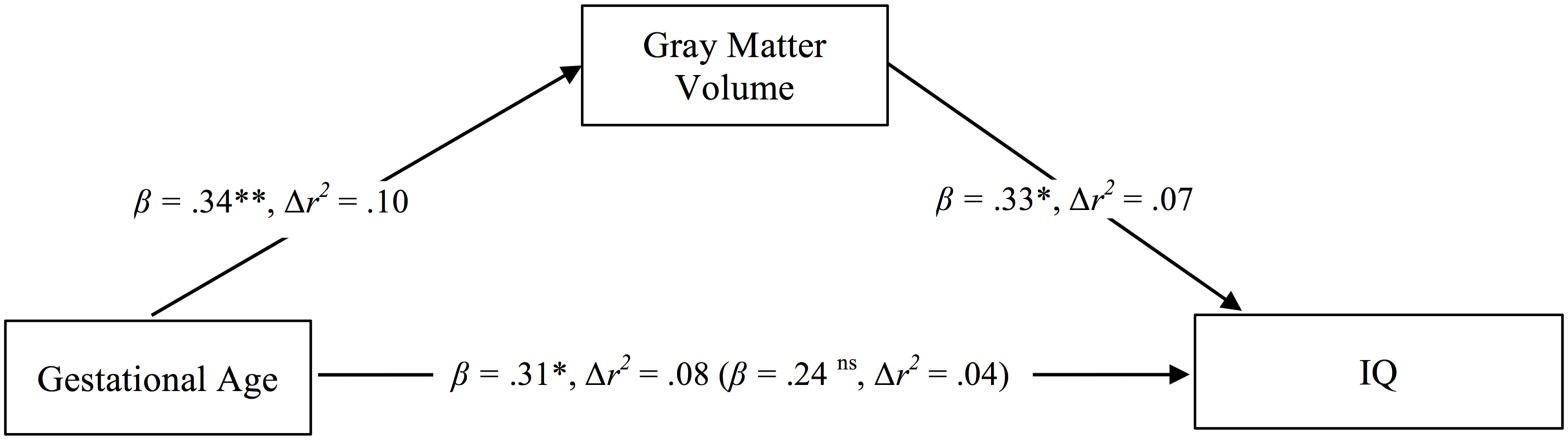
Mediation of the relationship between gestational age and IQ by GMV in preterm children (gestational age range: 24–32 completed weeks’ gestation). The direct effect predicting full scale IQ by GA controlling GMV volume is displayed in brackets. Coefficients are standardized regression coefficients controlled for age, sex, and maternal education. Explained variance (Δ*r*) is derived from a model that entered the predictor in a separate step holding covariates constant. * P < 0.05, ** P < 0.01.

Mediation analysis according to Baron and Kenny [29] is presented in Fig 3. Multiple regression analysis revealed that among preterm children GA (entered to the model as a continuous variable; range: 24–32 completed weeks’ gestation) was associated with GMV (*β* = 0.34, *t* = 3.04, *P* = 0.004, Δ*r*^*2*^ = .10 controlling age, sex, and maternal education). GMV was associated with full scale IQ (*β* = 0.33, *t* = 3.01, *P* = 0.003, Δ*r*^*2*^ = .07 controlling covariates). GA was associated with full scale IQ (*β* = 0.31, *t* = 2.25, *P* = 0.029, Δ*r*^*2*^ = .08 controlling covariates). However, this association was attenuated when GMV was controlled (*β* = 0.24, *t* = 1.64, *P* = 0.11, Δ*r*^*2*^ = .04), which indicates partial mediation of the relationship between GA and IQ by GMV.

Additional multiple regression analyses tested whether the regional cortical GMV differences in the superior and middle temporal gyrus of the right hemisphere that were found in children born 24–27 completed weeks’ gestation and 28–29 completed weeks’ gestation compared to term born children was associated with full scale IQ and the WISC-IV index scores. No significant association of these regions with cognitive functions could be revealed (P > 0.05; data not shown).

## Discussion

The aim of the present study was to assess the relationship between GA and GMV, WMV, and cognitive functions in preterm born children during school age who are at low risk (i.e., being born AGA and not exposed to major complications in the first year of life). In particular, we aimed at testing whether the relationship between GA and outcomes is linear or whether it follows a threshold model. The findings reveal a clear decline of GMV, WMV, and cognitive functions with GA among the groups of preterm children. Children born 24–27 completed weeks’ gestation showed decreased GMV, WMV, and cognitive functions compared to children born 30 completed weeks’ gestation and later. Decreased brain volume and cognitive functions in the group of children born before 28 completed weeks’ gestation is consistent with previous research [9,12,18,30,31]. The more GA is below a certain threshold, distress for the child and treatment requirements increase disproportionately possibly leading to subsequent alterations in brain development [32]. Mediation analysis showed that brain volume partially explained the association between GA and cognitive functions in the present sample of preterm children (GA range: 24–32 completed weeks’ gestation), which is also consistent with earlier research [9,12,19,25,33].

The preterm children born 30–31 and 32 completed weeks’ gestation did not show decrements in brain volume or cognitive functions compared to term born children and thus might be less affected by premature birth than it was previously believed [20,21,22,23,24]. As children born in the range of 30–32 completed weeks’ gestation constitute a considerable percentage of children who are nowadays entering the educational system with figures for e.g. Germany and the USA ranging between 0.7% and 1.4% of all children [25,26], this knowledge is of important prognostic value for parents, pediatricians, and educational services [25,26,32]. In contrast to earlier reports that indicated a linear relationship of brain volume across the full range of GA the present study only included children who were born AGA [19,20]. For children born 30–32 completed weeks’ gestation intrauterine growth restriction may be a decisive factor for brain and cognitive development. In line with this interpretation, recent research from the EPIPAGE study has shown that preterm children with restricted pre- and postnatal growth showed decreased cognitive and school performance [24]. One alternative explanation why brain volume and cognitive functions were not decreased in this group involves improvements in neonatal intensive treatment for very preterm children. The children studied here were born between 1998–2006 and treated in a highly specialized neonatal intensive care unit, which is different from many of the earlier neuroimaging studies on very preterm children. Though speculative, it is possible that particularly the group of children born 30–32 completed weeks’ gestation with low risk (no complications and born AGA) benefited from improved care. Between 1996 and 2008 there was a significant change in neonatal intensive treatment for very preterm children in Switzerland involving a marked increase in the use of antenatal corticosteroids, surfactant treatment, and the use of CPAP [32], which may also lead to improved long-term outcomes in this group.

Regarding regional reduction in GMV children born 24–27 completed weeks’ gestation and 28–29 completed weeks’ gestation both showed reduction in the superior and middle temporal gyrus of the right hemisphere compared to term born children, which is consistent with previous research [9]. This regional reduction of GMV was, however, not correlated with cognitive functions. Generally, the temporal lobes are involved in several tasks including integration of verbal and non-verbal memory, audio-visual association as well as object and face recognition while particularly the left hemisphere is involved in language recognition and production [33]. It may be hypothesized that GMV loss in the right hemisphere is due to compensatory processes of plasticity in the left temporal lobe that guarantee language networks to develop in disfavour of the right hemisphere [34]. While the group of children born 30–31 completed weeks’ gestation did not show any regional reduction compared to term born children, the group born 32 completed weeks’ gestation (moderately preterm children) showed a reduction in a small cluster in the left anterior insula. No regional increases in GMV were found in any of the groups of preterm children compared to those born at term.

As a limitation to the study, only a minority of all very preterm children born between 1998 and 2006 and treated in the study center could be recruited in the present study. Moreover, participating preterm children had higher birth weight than non-participants, which was due to selective inclusion of children who were AGA and without major complications. As a further limitation, our findings cannot be generalized to very preterm children born SGA or with major postnatal complications. Furthermore, the sizes of the subgroups of preterm children studied here were rather small, limiting the statistical power. Future studies addressing the question whether the relationship of gestational age and brain volume is linear or non-linear may include a larger number of very preterm children in these subgroups of GA, which might be achieved by pooling data from more than one cohort. Finally, potential non-representativeness of the term born control group might affect the findings. While we adjusted for possible confounders, we cannot completely rule out bias originating from the recruitment procedure of the control group.

## Conclusions

In conclusion, the present study informs that school age children born extremely preterm (i.e. before 28 completed weeks’ gestation) show decrements in overall and regional GMV, in overall WMV, and in cognitive functions. By contrast, children born 30 completed weeks’ gestation or later, who are at low risk (i.e. absence of major complications in the first year of life, born AGA), may be less strongly affected by decreased GMV, WMV, and cognitive functions than previously thought. This may indicate that the relationship of GA with brain volume and cognitive functions follows a threshold model with more evident effects below 28 completed weeks’ gestation.

## Supporting information

S1 Table Polynomial contrasts and pairwise comparisons between five gestational age groups (*P*-values) based on 1000 bootstrap samples and adjusted for age, sex, and maternal education. ^1^ Polynomial contrasts, ^2^ IQ score normative mean = 100 (SD = 15). GMV, Gray Matter Volume; WMV, White Matter Volume; WISC-IV, Wechsler Intelligence Scale for Children^®^–Fourth Edition. 24–27 completed weeks’ gestation: n = 10, 28–29 completed weeks’ gestation: n = 14, 30–31 completed weeks’ gestation: n = 19, 32 completed weeks’ gestation: n = 14, Term born: n = 49.
(DOCX)
