# Supplementary material for: Effects of gestational age on brain volume and cognitive functions in generally healthy very preterm born children during school-age: A voxel-based morphometry study
Source: PLoS One. 2017 Aug 29;12(8):e0183519. doi: 10.1371/journal.pone.0183519 (PMC5574554; doi:10.1371/journal.pone.0183519)
Supplement: S1 Table — 1 Polynomial contrasts, 2 IQ score normative mean = 100 (SD = 15). GMV, Gray Matter Volume; WMV, White Matter Volume; WISC-IV, Wechsler Intelligence Scale for Children®–Fourth Edition. 24–27 completed weeks’ gestation: n = 10, 28–29 completed weeks’ gestation: n = 14, 30–31 completed weeks’ gestation: n = 19, 32 completed weeks’ gestation: n = 14, Term born: n = 49. (DOCX) [file pone.0183519.s001.docx]

**S1 Table. Polynomial contrasts and pairwise comparisons between five gestational age groups (*P*-values) based on 1000 bootstrap samples and adjusted for age, sex, and maternal education.**

|  | **Linear trend^1^** | **Quadratic trend^1^** | **Cubic trend^1^** | **24-27 weeks**  **vs**  **28-29 weeks** | **24-27 weeks**  **vs**  **30-31 weeks** | **24-27 weeks**  **vs**  **32 weeks** | **24-27 weeks**  **vs**  **Term born** | **28-29 weeks vs**  **30-31 weeks** | **28-29 weeks vs**  **32 weeks** | **28-29 weeks vs**  **Term born** | **30-31 weeks vs**  **32 weeks** | **30-31 weeks vs**  **Term born** | **32 weeks**  **vs**  **Term born** |
| --- | --- | --- | --- | --- | --- | --- | --- | --- | --- | --- | --- | --- | --- |
|  | ***P*** | ***P*** | ***P*** | ***P*** | ***P*** | ***P*** | ***P*** | ***P*** | ***P*** | ***P*** | ***P*** | ***P*** | ***P*** |
| **GMV (ml)** | 0.013 | 0.115 | 0.199 | 0.774 | 0.012 | 0.018 | 0.038 | 0.020 | 0.028 | 0.090 | 0.833 | 0.400 | 0.346 |
| **WMV (ml)** | 0.006 | 0.006 | 0.420 | 0.070 | 0.002 | 0.001 | 0.013 | 0.247 | 0.077 | 0.679 | 0.381 | 0.366 | 0.073 |
| **Full Scale IQ (WISC-IV)^2^** | 0.002 | 0.044 | 0.367 | 0.394 | 0.025 | 0.006 | 0.028 | 0.128 | 0.036 | 0.202 | 0.499 | 0.697 | 0.205 |
| **Verbal Comprehension^2^** | 0.164 | 0.078 | 0.447 | 0.238 | 0.087 | 0.003 | 0.174 | 0.848 | 0.207 | 0.784 | 0.193 | 0.567 | 0.058 |
| **Reasoning^2^** | 0.020 | 0.213 | 0.628 | 0.402 | 0.213 | 0.217 | 0.148 | 0.502 | 0.538 | 0.323 | 0.974 | 0.801 | 0.773 |
| **Working Memory^2^** | 0.083 | 0.139 | 0.313 | 0.689 | 0.057 | 0.057 | 0.144 | 0.117 | 0.108 | 0.381 | 0.757 | 0.359 | 0.254 |
| **Processing speed^2^** | 0.001 | 0.121 | 0.902 | 0.296 | 0.042 | 0.012 | 0.010 | 0.202 | 0.120 | 0.091 | 0.791 | 0.806 | 0.963 |

^1^ Polynomial contrasts

^2^ IQ score normative mean = 100 (SD = 15).

GMV, Gray Matter Volume; WMV, White Matter Volume; WISC-IV, Wechsler Intelligence Scale for Children®–Fourth Edition.

24-27 completed weeks’ gestation: n = 10

28-29 completed weeks’ gestation: n = 14

30-31 completed weeks’ gestation: n = 19

32 completed weeks’ gestation: n = 14

Term born: n = 49
